# Guideline for the use of immunobiologicals in chronic rhinosinusitis with nasal polyps (CRSwNP) in Brazil

**DOI:** 10.1016/j.bjorl.2021.03.003

**Published:** 2021-04-03

**Authors:** Wilma T. Anselmo-Lima, Edwin Tamashiro, Fabrizio R. Romano, Marcel M. Miyake, Renato Roithmann, Eduardo M. Kosugi, Márcio Nakanishi, Marco A. Fornazieri, Thiago F.P. Bezerra, João F. Mello, Marcus M. Lessa, Richard L. Voegels, Otávio B. Piltcher, Eulalia Sakano, Fabiana C.P. Valera

**Affiliations:** a Universidade de São Paulo (USP), Faculdade de Medicina de Ribeirão Preto (FMRP), Ribeirão Preto, SP, Brazil; b Universidade de São Paulo (USP), Faculdade de Medicina (FM), São Paulo, SP, Brazil; c Hospital da Santa Casa de Misericórdia de São Paulo, São Paulo, SP, Brazil; d Universidade Luterana do Brasil, Canoas, RS, Brazil; e Universidade Federal de São Paulo (UNIFESP), Escola Paulista de Medicina (EPM), São Paulo, SP, Brazil; f Universidade de Brasília (UnB), Faculdade de Medicina, Brasília, DF, Brazil; g Universidade Estadual de Londrina (UEL), Londrina, PR, Brazil; h Pontifícia Universidade Católica do Paraná (PUC-PR), Curitiba, PR, Brazil; i Universidade Federal de Pernambuco (UFPE), Recife, PE, Brazil; j Universidade Federal da Bahia, Faculdade de Medicina, Salvador, BA, Brazil; k Universidade Federal do Rio Grande do Sul (UFRGS), Faculdade de Medicina (FAMED), Porto Alegre, RS, Brazil; l Universidade Estadual de Campinas (UNICAMP), Faculdade de Medicina, São Paulo, SP, Brazil

**Keywords:** Immunobiologicals, Monoclonal antibody, Chronic rhinosinusitis, Nasal polyps

## Abstract

**Introduction:**

Severe uncontrolled chronic rhinosinusitis with nasal polyps has a negative impact on an individual’s quality of life. Therefore, new biologics have emerged for use in specific phenotypes of chronic rhinosinusitis, changing the paradigms of its treatment.

**Objective:**

To review the current status of biologic treatment indications in chronic rhinosinusitis.

**Methods:**

The Brazilian Academy of Rhinology brought together different specialists to suggest a course of action, considering its particularities and aspects related to the national reality.

**Results:**

Of particular interest for decision making will be the identification of subgroups of patients refractory to pre-existing treatment options and the construction of a strategy that improves their quality of life, with the best cost-benefit ratio.

**Conclusion:**

The use of biologics is a valid option for treatment in more severe cases. This strategy must be better understood and improved in the future, with more studies and greater clinical experience.

## Highlights

• The use of immunobiologicals is an option for the management of patients with chronic rinosinusitis with nasal polyps (CRSwNP) refractory to conventional treatment.
• The guideline lists the main drugs currently available in Brazil, their indications, and recommendations for use.

## Introduction

Currently, there is a significant increase in the publication of articles on the use of new immunobiologicals in specific phenotypes of chronic rhinosinusitis (CRS), changing the treatment paradigms in cases refractory to conventional therapy.^1^ In view of the rapid and growing supply of these new drugs, the Brazilian Academy of Rhinology brought together different specialists to review the current status of the indications for immunobiologicals in CRS, considering their particularities and aspects related to the national reality. Today, the immunobiologicals intended for chronic rhinosinusitis with nasal polyps (CRSwN) treatment act exclusively on type 2 inflammation.

Type 2 inflammation, formerly called T-helper type 2 (Th2) inflammation, is orchestrated by inflammatory mediators produced by Th2 cells, such as the cytokines IL-4, IL-5, IL-9 and IL-13, having eosinophil as the main cell marker, in addition to the high presence of local or circulating IgE. With the subsequent identification of the participation of other non-Th2 cells capable of producing the same cytokine profile, such as type 2 innate lymphoid ells (ILC2), the inflammation became known as type 2.

An essential aspect was the recognition that CRS encompasses a range of situations with different pathophysiological bases, although they have common clinical characteristics. Consequently, specific forms of CRS have different prognosis and responses to a given treatment. A recent multicenter study revealed that 35% of CRSwNP patients had recurrence within 6 months.^2^ In the quest to identify the factors potentially associated with worsening outcomes, endotyping, through the use of biomarkers, can help identify which patients have type 2 inflammation and who would respond best to these new treatments.^2^, ^3^

## Type 2 interleukins and their effects on CRS

IL-5 is a cytokine that controls the differentiation and maturation of eosinophils in the bone marrow, in addition to inducing activation and increasing their survival in the tissue, reducing the degree of apoptosis.^3^, ^4^, ^5^ The effects of IL-4 include the differentiation of T lymphocytes into Th2, induction of B lymphocytes for IgE production, chemotaxis for eosinophils and recruitment and activation of mast cells and basophils.^3^, ^4^, ^5^ IL-13 is chemotactic for eosinophils, induces B lymphocytes to produce IgE and activates mast cells and basophils. In addition, it induces mucus secretion, goblet cell hyperplasia, and collagen production.^3^, ^4^, ^5^ IL-33 is also a mediator of type 2 inflammation. It binds to a surface receptor on Th2, ILC2 lymphocytes, basophils, eosinophils, mast cells, and dendritic cells, among others, activating inflammation in the airways. Direct exposure of the airway epithelium to S. aureus increases the expression of IL-33 and TSLP, which induce the production of cytokines such as IL-5 and IL-13, playing a role in the onset and/or maintenance of type 2 inflammation in CRSwNP.^2^, ^5^

## Immunobiologicals

Several immunobiological products are being studied for use in respiratory diseases, such as anti-IgE (omalizumab), anti-IL-5 (mepolizumab, reslizumab, benralizumab), anti-IL-4, and anti-IL-13 (dupilumab), among others.

## Anti-IgE (omalizumab)

Omalizumab is an anti-IgE monoclonal antibody initially approved in 2003 by the FDA for the treatment of moderate-persistent allergic asthma, not controlled with inhaled corticosteroids, being the first immunobiological agente used for type 2 inflammatory diseases.^6^, ^7^, ^8^, ^9^ Currently, it is indicated also for patients with chronic urticaria, and very recently, for patients with CRSwNP.^10^ For asthma, studies have shown improvement in disease control, reduction in the number of exacerbations and the need for oral corticosteroids as well as the use of rescue medications.^7^, ^10^, ^11^, ^12^ After subcutaneous administration, omalizumab is slowly absorbed, reaching the peak of serum concentrations after an average of 7–8 days, with a terminal half-life of 26 days.^13^, ^14^

### Review of the use in CRS

Given the high concentrations of mucosal IgE in the nasal polyp tissue and its relevance to the severity of the disease and comorbidity, its inhibition may have an impact on patients with CRSwNP.^15^

In a randomized, double-blind, placebo-controlled phase II study with patients with CRSwNP and associated asthma, Gevaert et al. selected patients to receive 4–8 subcutaneous doses of omalizumab (n = 16) or placebo (n = 8) for 16 weeks.^16^ There was a significant reduction in the nasal polyp endoscopic score and in the Lund-MacKay tomographic score in the omalizumab group compared to the placebo group. In addition, omalizumab had a significantly greater beneficial effect on sinunasal symptoms, including nasal congestion, anterior rhinorrhea and loss of smell, as well as lower airway symptoms, including wheezing and dyspnoea. Finally, omalizumab was also associated with better quality of life scores in patients with CRSwNP and asthma.^8^, ^10^, ^16^

Pinto et al., in another randomized, double-blind, placebo-controlled clinical trial of refractory CRSwNP, randomized the patients to receive omalizumab or placebo for 6 months.^17^ Treatment with omalizumab was associated with a significant improvement in quality of life (SNOT-20) at various time intervals, including 3, 5 and 6 months of followup compared to the baseline, whereas there were no significant changes in the control group.^8^, ^17^

Rivero and Liang, in a systematic review including studies of anti-IgE therapy, found no statistically significant reduction in the nasal polyp score compared to the placebo group, although they did tend to improve.^18^ However, in the post hoc analysis, the authors observed that patients who had concomitant severe asthma experienced a statistically significant reduction in the nasal polyp score. They concluded that anti-IgE therapy reduces the nasal polyps score in patients with associated severe asthma.^18^

In another systematic review, Tsetsos et al.^19^ compared the results of randomized clinical trials performed by Gevaert et al.^16^ and by Pinto et al.^17^ assessing the efficacy of omalizumab in patients with CRSwNP and associated asthma. Clinical improvement was measured in both clinical trials through the total nasal polyp score, sinus opacification at paranasal sinus tomography, and measuments of quality of life, peak nasal airflow, nasal inspiratory flow, and sense of smell (UPSIT).^10^, ^19^ While the clinical trial conducted by Pinto et al. found no statistically significant changes in any of the categories cited, the study by Gevaert et al. found significant improvement in all measurements, except for nasal airflow and smell. It should also be noted that the study by Gevaert et al. had as limitations the inclusion of a limited number of participants (n = 24), the highest baseline eosinophilic inflammation in subjects treated with placebo (despite randomization), and the high dropout rate in the placebo group (50%). Pinto et al. also highlighted the limitation regarding the number of participants enrolled in their study, emphasizing the need for a trial with a larger number of patients. Two other systematic reviews also reported the need for further evaluation of the effectiveness of anti-IgE therapy in these patients.^20^, ^21^

More recently, Gevaert et al. published the results of the Phase III POLYP 1 and 2 studies.^22^ They demonstrated that patients treated with omalizumab and intranasal mometasone achieved statistically significant improvements in nasal polyp score and daily nasal congestion score compared to standard therapy (placebo and intranasal mometasone). This difference in parameters was observed from the first assessment in the 4th week to the 24th week. Among the secondary objectives, improvements were observed in the nasosinusal quality of life score (sino-nasal outcome test-22 – SNOT-22), in the University of Pennsylvania smell identification test (UPSIT), in the total nasal symptom score (TNSS), and in the specific complaint of sense of smell. In addition, reductions in posterior and anterior rhinorrhea were observed. In these studies, omalizumab was generally well tolerated and its safety profile was consistent with previous studies.^22^

Omalizumab has also been shown to be clinically beneficial in patients with moderate to severe asthma and associated allergic fungal rhinosinusitis (AFRS).^23^ Mostafa et al.^13^ compared a single postoperative injection of omalizumab (at a dose of 150 mg; n = 10) with intranasal corticosteroid spray (budesonide or mometasone furoate, 100 μg twice daily, n = 10) for 6 months in patients with AFRS, with patients being evaluated at an interval of 4 weeks for 6 months. Both treatments were effective at the end of 24 weeks of followup, but the omalizumab group showed a more significant endoscopic and clinical response, particularly in allergic symptoms such as sneezing, itching and nasal discharge. There were no significant side effects in either group.^2^

Regarding the choice of therapy with anti-IL-5, the often proposed differentiation between non-allergic patients with nasal polyps and elevated blood eosinophils and patients allergic to anti-IgE is not supported by evidence. In comparison, it is worth mentioning that omalizumab worked at least as well in non-allergic individuals as it did in allergic individuals. Omalizumab is known to decrease free IgE antibodies, but it is not yet clear which biomarker is important for its clinical effect in nasal polyps. No biomarkers currently used, such as blood eosinophils or total serum IgE, have been shown to assist in the selection or prediction of responses to immunobiologicals.^24^ In the study by Gevaert et al., there was improvement in the scores of total nasal polyps, opacification of paranasal sinuses and nasal symptoms, including smell, in both allergic and non-allergic individuals.^16^ In addition, omalizumab significantly improved asthma symptoms and quality of life, regardless of the presence of allergy.^25^ Recent observations that demonstrate its efficacy in patients with non-allergic asthma support these findings.^2^

According to the medicine regulatory agencies, such as the European Medicines Agency (EMA) in Europe, Food and Drug Administration (FDA) in the United States, and Agência Nacional de Vigilância Sanitária (ANVISA) in Brazil, omalizumab is indicated for persistent moderate to severe allergic asthma uncontrolled with inhaled corticosteroids in adults and children (over 6 years of age), as additional therapy in adult and pediatric patients (over 12 years of age) with spontaneous chronic urticaria refractory to treatment with H1 antihistamines,^11^ and for adult patients (over 18 years old) with CRSwNP refractory to conventional treatment, as complementary therapy to intranasal corticosteroids.^11^

Omalizumab is administered by subcutaneous injection every 2–4 weeks, with a dosage based on the baseline serum IgE level (IU/mL), measured before starting treatment, and body weight (kg).^10^

Because omalizumab does not bind to IgE, which is already bound to receptors on effector cells, the onset of clinical activity is somewhat delayed. Clinical trials have shown benefits over placebo after 4 weeks of therapy, although the maximum effects may take longer.^9^ Therefore, systemic or inhaled corticosteroid therapy should not be stopped immediately after starting therapy with omalizumab. Corticosteroid dosage reduction should be attempted gradually over several weeks under medical guidance.^9^

Omalizumab was generally well tolerated in adolescents and adults with allergic asthma in clinical trials.^9^ It should be administered in controlled center clinic, due to a 0.2% risk of anaphylaxis.^10^ Previously, there were concerns that omalizumab was associated with malignancy; however, this risk was discarded a after a prospective cohort study (EXCELS).^26^ In 2014, the FDA added cardiovascular risks to the omalizumab label. The most common adverse events were upper respiratory tract infection, injection site reaction and headache, although the incidence of these events was similar to that of placebo.^9^, ^10^ The most common local reactions include bruising, redness, heat and itching, most often within 1 h after taking the medication, but the frequency of these reactions generally decreases with continued use of the drug.^9^ Although causal relationships have not been demonstrated, diseases similar to eosinophilic vasculitis, such as eosinophilic granulomatosis with polyangiitis (EGPA), have also been reported with omalizumab, usually with concomitant reduction in corticosteroid therapy.^18^ Finally, opportunistic infection with herpes zoster and helminth infections are theoretical risks, and monitoring of these infections should be carried out at the physician’s discretion.^18^

## Anti-IL-5 (mepolizumab, reslizumab and benralizumab)

IL-5 is a cytokine that plays a key role in the activation, differentiation, chemotaxis, and survival of eosinophils.^27^, ^28^ Together with the cytokines IL-4 and IL-13, it is a typical marker of type 2 inflammatory response, and it is increased in most patients with CRSwNP. However, in some populations such as the Chinese, the levels are not as high as in the Caucasian populations.^29^

Mepolizumab and reslizumab are IL-5 antagonist monoclonal antibodies. They bind to IL-5 and inhibit its signaling, consequently reducing the production, maturation, and survival of eosinophils.^30^, ^31^ Mepolizumab inhibits the bioactivity of IL-5 with nanomolar potency by blocking the binding of IL-5 to the alpha chain of the receptor complex of that cytokine expressed on the cell surface of the eosinophil, in order to inhibit IL-5 signaling and reduce the production and survival of eosinophils.^32^ Reslizumab specifically binds to IL-5 and interferes with the binding of IL-5 to its cell surface receptor.^33^

Benralizumab is a monoclonal antibody directed at the IL-5 receptor. It binds to the alpha subunit of the human Interleukin-5 receptor (IL-5Rα) with high affinity and specificity. The IL-5 receptor is expressed specifically on the surface of eosinophils and basophils. The absence of fucose sugar in the Fc domain of benralizumab results in a high affinity for the FcɣRIII receptors of effector immune cells, such as natural killer cells, leading to apoptosis of eosinophils and basophils by increasing cellular cytotoxicity.^34^

### Review of the use in CRS

To date, there are few randomized clinical trials (RCTs) evaluating these immunobiologicals in the treatment of CRS patients. There are two RCTs of benralizumab, one of which is in progress,^35^ three of mepolizumab (one still in progress),^36^ and one of reslizumab, in addition to two systematic reviews on the use of these immunobiologicals for CRSwNP treatment.^37^ All of them demonstrate that the different anti-IL-5 immunobiologicals promote symptom improvement compared to the placebo group in the various parameters evaluated, among them: quality of life, nasal obstruction, need to use systemic corticosteroids to relieve nasal symptoms, sense of smell, size of polyps, opacification on computed tomography, and need for CRSwNP surgery.

Gevaert et al.^37^ were the first to investigate anti-IL-5 therapy for CRSwNP. In that study, the improvement in the CRSwNP score was approximately 50% in the reslizumab groups. The authors correlated better results with the pretreatment of higher levels of nasal IL-5. This same study showed eosinophilia rebound after the end of treatments at different times according to the dose administered.

On the other hand, studies of mepolizumab did not show a rebound effect on the serum eosinophil count after discontinuing anti-IL-5 therapy. Although mepolizumab therapy also shows globally benefits regarding polyp size and tomographic extension scores, the percentages of improvement also did not exceed 60% of the treated patients.^29^

Bachert et al.,^38^ in the largest RCT available to date, investigated whether anti-IL5 therapy would reduce the need for surgical treatments. This answer becomes even more relevant in view of the costs involved with surgical treatments and with the immunobiologicals themselves. The authors identified a significant reduction in the mepolizumab-treated group, compared to the placebo group in terms of surgical indication based on pre-established clinical criteria: a 30% reduction in surgical need compared to 10% of patients in the placebo arm over a period of 9 weeks of evaluation. However, the duration of the benefits conferred by this form of treatment has yet to be assessed.

Some studies are ongoing^35^, ^36^ and more data will soon be available;^39^ for example, for benrazilumab with a significant number of patients with severe CRSwNP for a period of 56 weeks and mepolizumab for 52 weeks. These studies may collaborate in defining the real role and indication of these immunobiologicals in the treatment of CRSwNP. The available literature including the described RCTs and two systematic reviews^40^, ^41^ have so far demonstrated that the blockade of the inflammatory response related to IL-5 is clearly proven to decrease systemic and nasal eosinophilia. However, studies involving eosinophilic CRS still need to be performed to predict which subgroups of patients will show better responses, in order to minimize the waste of resources and maximize the effects of anti-IL-5 therapy and define the real role of these immunobiologicals in CRS.

Mepolizumab is indicated for treating patients 12 years of age and older with severe eosinophilic asthma.^30^ Also, it is indicated for treating relapsing or refractory EGPA.^32^ Benralizumab is indicated for treating patients 12 years of age and older with severe eosinophilic asthma.^35^ Reslizumab is indicated for maintenance therapy of severe asthma associated with other drugs in patients over 18 years of age.^35^

Mepolizumab is used as a subcutaneous injection. For asthma, the recommended dosage is 100 mg every 4 weeks. In children aged 6–11 years, 40 mg should be administered every 4 weeks.^37^ The dosage for EGPA is 300 mg every 4 weeks.^32^

Reslizumab is used as an intravenous infusion every 4 weeks, at a dosage of 3 mg/kg.^31^

Benralizumab is administered subcutaneously at a dosage of 30 mg every 4 weeks for the first three doses, and thereafter every 8 weeks.^33^

The safety and tolerance of anti-IL-5 are already established.^37^, ^38^, ^39^ Anti-IL-5 drugs are safe and well tolerated, with the most common side effects being headache, reaction at the injection site, back pain, and fatigue.^37^ In a study of mepolizumab in severe CRSwNP, the most common side effects were: pharyngitis, increased serum creatine phosphokinase, and myalgia.^28^, ^38^ Reslizumab was considered safe and well tolerated in CRSwNP patients.^33^ Side effects of benralizumab are headache, pharyngitis, and reaction at the injection site.^40^, ^41^

A concern related to the use of anti-IL-5 was the reduced host defense.^38^ However, in clinical trials of mepolizumab and benralizumab used for 1 year, the frequency of upper respiratory tract infections was less than that of the placebo group.^40^, ^41^ Another concern was related to the association of anti-IL-5 with malignant tumors. However, the incidence rate of malignancy was similar to that seen in the placebo group.^40^

Systemic reactions with the use of mepolizumab and benralizumab were hypersensitivity reactions in 2% and 1%–3%, respectively. Headache occurred more frequently when using mepolizumab (20% higher compared to placebo group) and benralizumab (7%–9%) compared to the placebo group (5%–7%).^41^

## Anti-IL-4/IL-13 (dupilumab)

Dupilumab is the first immunobiological with indication of specific use for CRSwNP authorized by the main international regulatory agencies (FDA and EMA in 2019)^42^, ^43^ and in Brazil (ANVISA in 2020).

IL-4 and IL-13 are potent mediators of type 2 inflammation, sharing the same receptor and signaling pathways, being involved in IgE synthesis, recruitment of eosinophils to inflamed tissue, mucus secretion, and airway remodeling. IL-4 is one of the main differentiating factors for the Th2 response, in addition to inducing the production of type 2 cytokines and chemokines such as IL-5, IL-9, IL-13, TARC and eotaxin. Additionally, IL-4 and IL-13 are responsible for changing the isotype of B cells for IgE^1^ production. Dupilumab is a recombinant human IgG4 monoclonal antibody directed against the interleukin-4 α-receptor (IL-4Rα). Its blockade inhibits IL-4/IL-13 signaling, decreasing the type 2 immune response.^44^

### Review of the use in CRS

Bachert et al.^45^ published a double-blind placebo controlled clinical trial that randomized 60 adults with CRSwNP into two groups. After four weeks of initial mometasone treatment, patients were randomized to subcutaneous dupilumab (initial dose of 600 mg followed by 15 weekly doses of 300 mg) or corresponding placebo for 16 weeks.Patients treated with dupilumab had a significant improvement in quality of life, intensity of rhinosinusitis, nasal obstruction, sense of smell, nasal polyp size, tomographic and asthma scores (clinical control and lung function).

Bachert et al.^46^ published the results of two randomized, double-blind, multicenter, placebo-controlled studies that evaluated dupilumab added to standard treatment in adults with severe CRSwNP. In the LIBERTY NP SINUS-24 study, patients were randomized 1:1 for 24 weeks with dupilumab 300 mg or placebo every two weeks. In LIBERTY NP SINUS-52, patients were randomized 1:1:1 with: a) 52 weeks with dupilumab 300 mg every two weeks, b) 24 weeks with dupilumab 300 mg every two weeks, and then 28 weeks with dupilumab 300 mg every four weeks or; c) 52 weeks with placebo every two weeks. In both studies, dupilumab significantly improved quality of life, intensity of rhinosinusitis, nasal obstruction, sense of smell, polyps size, nasal endoscopy, and lung function. It is interesting to note that the improvement in sense of smell is observed regardless of whether the patient has undergone previous surgery.^13^ These results were also supported in several post-hoc analyzes^45^, ^47^, ^48^, ^49^ as well as in a recent systematic review.^50^

Dupilumab also reduced the concentrations of biomarkers of eosinophilic inflammation: serum IgE, eotaxin-3, periostin and TARC; IgE, eosinophilic cationic protein, eotaxin-2, eotaxin-3, PARC, IL-13, periostin, and tissue IL-5.^51^

There is a proven beneficial effect in CRSwNP associated with AERD,^52^, ^53^ and anecdotal evidence in allergic fungal rhinosinusitis.^54^ The relative risk for further surgery after starting dupilumab is considerably reduced.^46^, ^47^ When combined with intranasal corticosteroids, it reduces sick leave and improves productivity at work.^47^

Dupilumab is indicated as a complementary treatment for CRSwNP in adults who have failed previous treatments, or who are intolerant or with contraindications to oral corticosteroids and/or surgery. It should not be used to treat patients with acute bronchospasm or asthmatic condition or patients with helminth infections; these conditions should be treated prior to starting treatment with Dupilumab.^55^

The dosage of dupilumab for CRSwNP, administered subcutaneously, is 300 mg, which is usually administered for the first time in the clinic, and thereafter, every 2 weeks, at home. It can be self-administered by the patient, administered by a health professional or a caregiver. If the patient forgets to administer a dose, it should be administered as soon as possible. After that, the patient should resume the regularly established dosing regimen.^55^

Contrary to what was seen in patients with atopic dermatitis, patients with asthma or CRSwNP did not present conjunctivitis as an adverse event.^56^ The most common adverse events (more frequent than placebo) were: nasopharyngitis, worsening of nasal polyps and asthma, headache, epistaxis, and erythema at the injection site.^55^

This medication should not be used by pregnant or lactating women without medical advice. Safety and efficacy in pediatric patients, under the age of 18, have not been established.^56^

## Future immunobiologicals for eosinophilic chronic rhinosinusitis

While the current commercially available immunobiologicals focus on the type 2 adaptive immune response (mainly in the cytokines IL-5, IL-4 and IL-13, in addition to IgE), new potential immunobiologicals have been developed, focusing on the innate immune response.

Among the potential innate immunity cytokines as therapeutic targets, two stand out in the literature: IL-33 and TSLP. Both are produced in the epithelium, and have a more comprehensive ability to induce an eosinophilic response.

Of the new anti-IL-33 (etokimab)^57^, ^58^ and anti-TSLP (tezepelumab) immunobiologicals,^59^, ^60^, ^61^, ^62^, ^63^ only etokimab has an ongoing study for CRSwNP (Clinicaltrials.gov Identifier: NCT03614923).

## Immunobiologicals for secondary CRS

### EGPA (eosinophilic granulomatosis with polyangiitis)

The most studied immunobiologicals for EGPA, also known as Churg-Strauss syndrome, are the drugs that act on IL-5, either directly (mepolizumab) or through its receptor (benralizumab).^64^, ^65^, ^66^, ^67^ It is worth mentioning that, to date, mepolizumab is the only one that has been approved by the regulatory agencies (FDA, EMA and ANVISA) for EGPA treatment.

Mepolizumab has been evaluated in two double-blind, randomized, multicentre phase 3 clinical studies. Wechsler et al.^64^ evaluated 136 patients with refractory EGPA who had been using systemic corticosteroids for at least 4 weeks, evaluating the effect of mepolizumab at a dose of 300 mg subcutaneously every 4 weeks for a total period of 52 weeks. In this study, participants using mepolizumab had a 16.74 times greater chance of disease remission at the end of the study, and 0.32 times less chance of having recurrences during the total evaluation period, both of which were significant. Additionally, patients receiving mepolizumab required a lower dose of prednisone or prednisolone on average (Odds Ratio: 0.20; *p* < 0.001). Interestingly, patients with more than 150 eosinophils/mm^3^ in peripheral blood had a response 26.10 times more robust than patients with less than 150 eosinophil/mm^3^.

In another multicenter double-blind randomized phase 3 study, Steinfeld et al.^65^ evaluated 136 patients, using the same dose and frequency as the previous study. They observed that 78% of the patients using mepolizumab and 32% of the placebo group had the clinical benefit, when considered criteria: Birmingham vasculitis activity score (BVAS) of 0 and use of corticosteroids at a dose lower than 4 mg/day OR reduction of corticosteroid dose in more than 50% OR absence of recurrence of symptoms). With less strict criteria (BVAS score of 0 and use of corticosteroids at less than 7.5 mg/day OR reduction in the dose of corticosteroids by more than 50% OR absence of recurrence of symptoms), the clinical benefit was reached by 87% of the mepolizumab group and 53% of the placebo group during the study.

In a study of real life data of EGPA patients dependent on the use of systemic corticosteroids, in which patients were treated with mepolizumab (100 or 300 mg every 4 weeks) or omalizumab, Canzian et al.^66^ observed that mepolizumab, in any of the prescribed doses, was better than omalizumab in reducing the use of corticosteroids to rescue symptom control, in remission after 12 months of use (78% vs 15%), and in therapeutic failures (8% vs 48%). The authors also reported that the remission rate was similar at different doses of mepolizumab. However, 2 patients experienced persistent symptoms at the dose of 100 mg, and benefited from increasing the dose to 300 mg.

Benralizumab is also being studied in EGPA patients, but only one open-label clinical study, with only 10 patients, has been published so far. In this article, Guntur et al.^67^ demonstrated that the concomitant use of benralizumab 30 mg was able to significantly reduce the dose of systemic corticosteroids. Double-blind, randomized studies, with a larger number of patients, are being carried out to confirm the ability of benralizumab to control the EGPA respiratory symptoms (ClinicalTrials.gov Identifier: NCT04157348, NCT03010436), as well as the use of reslizumab (ClinicalTrials. gov Identifier: NCT02947945).

In general, these drugs may lead to mild to moderate adverse events, the most common of which are headache, local reaction, back pain, fatigue, rhinorrhea, and nasal congestion. More serious adverse events are rare, with anaphylaxis being the most reported.^65^, ^67^

### GPA (granulomatosis with polyangiitis)

Rituximab (anti-CD20 antibody) is the most widely used immunobiological agent for patients with granulomatosis with polyangiitis (GPA), formerly known as Wegener's granulomatosis.^68^

In the main double-blind, randomized multicenter clinical study, Unizony et al.^68^ evaluated 197 patients, 148 with GPA, 48 with microscopic polyangiitis (MPA), and one undefined patient, who were divided into two groups: rituximab 375 mg/m2 intravenously per week for 4 weeks, or comparative treatment (cyclophosphamide and azathioprine). Patients using rituximab were 2.11 times more likely to experience clinical remission. The difference in response was even greater in the group refractory to treatment, with Odds Ratio of 3.57 at 6 months; 4.32 at 12 months, and 3.06 at 18 months.

Charles et al.^69^ evaluated 68 patients with GPA and 29 with long-term MAP in a double-blind, randomized, multicenter clinical study. All patients received rituximab during the first 28 weeks, and were in control of the disease at this time. Subsequently, patients were randomized to rituximab 500 mg every 6 months, for an additional 18 months, or placebo. Disease-free survival after 28 weeks of the second cycle was 96% in the rituximab group, versus 74% in the placebo group (Hazard Ratio 7.5; *p* < 0.01), showing a potential long-term use of this medication for GPA control.

Puéchal et al.^70^ used rituximab to induce GPA control in a cohort study involving 114 patients (65% with recurrent disease, 22% with difficult-to-control disease, and 13% with initial disease). All patients received rituximab at induction; in 90 of them, maintenance doses were necessary for symptom control (500 mg every 6 months, up to an accumulated dose of 2 g). Patients were followed for an average of 3.6 years, with disease-free survival of 85% after 2 years of follow-up. Serious infections occurred in 4.9% of patients/year, and more important adverse events occurred in 8.1%. Multivariate analysis showed that persistence of symptoms, presence of subglottic stenosis, otorhinolaryngological symptoms, and presence of skin lesions were all associated with a lower chance of remission.

Despite the studies presented above, two systematic reviews mention that there is still a low level of evidence on the benefit of using rituximab for GPA treatment.^71^, ^72^ An additional precaution is the risk of adverse events, including serious infections, which are more frequent with this immunobiological agent than with those previously mentioned.

### Sense of smell and immunobiologicals

Among the most promising effects of immunobiologicals on CRSwNP patients is improved olfaction. Through objective and validated tests, studies have observed clinically significant improvement in patients with anosmia, in mild hyposmia or even normosmia, after treatment with immunobiologicals.

For this reason, anosmia is an important criterion established by the main guidelines for the indication of CRSwNP treatment with immunobiologicals.^1^, ^73^ It is important to emphasize that the diagnosis of anosmia must be made by a validated psychophysical test, and not only through questionnaires that assess the patient’s perception of olfactory loss. The indication of immunobiologicals requires that the patient meets the loss of smell criterion, classified as severe hyposmia or anosmia, regardless of the test used. Mild to moderate hyposmia in CRSwNP tends to have a good resolution with the use of topical and systemic steroids.^74^

## Criteria for the indication of immunobiologicals in CRSwNP

The indication of immunobiologicals in patients with eCRSwNP needs to meet two criteria: having type 2 inflammation (Table 1) and having serious and uncontrolled disease (Table 2).

**Table 1:** Clinical and laboratory criteria suggestive of CRS with type 2 inflammation (eosinophilic CRSwNP).

**At least 3 of the clinical criteria below:**
Clinical history: Age at onset of symptoms between 30–50 years
Significant improvement in smell with oral corticosteroids
Adult-onset asthma
Aspirin or NSAID intolerance
Presence of bilateral nasal polyps and thick nasal mucus (allergic/eosinophilic mucin), preferably confirmed by nasal endoscopy
**And**
**At least 1 biomarker below:**
Tissue eosinophilia ≥10 cells/high-power field
Serum eosinophilia ≥250 cells/mcL
Total serum IgE ≥100 IU/mL

**Table 2:** Criteria for uncontrolled severe chronic rhinosinusitis.

**MANDATORY presence of the criterion:**
Persistence of symptoms after optimized clinical and surgical treatment
**Associated with the presence of at least 3 clinical criteria below:**
Moderate to severe nasal congestion measured by Visual Analogue Scale (VAS) ≥5
Severe hyposmia or anosmia measured by tests validated in Brazil
SNOT-22 > 35
Uncontrolled asthma
At least 2 courses of oral corticosteroids in one year
At least 1 previous nasal endoscopic surgery
**And 1 more of the two criteria:**
Presence of bilateral nasal polyps extending beyond the middle meatus, preferably confirmed by nasal endoscopy
Computed tomography with significant opacification – minimum Lund-Mackay score of 10

### Evaluation of the clinical response to an immunobiological agent

When an immunobiological agent is selected to treat severe uncontrolled CRSwNP, it is important to monitor the patient’s response to the drug. To avoid inappropriate treatment and unnecessary costs, the response to treatment should be reassessed every 4–6 months (Table 3). This period is considered more than adequate to observe the clinical response to the prescribed medication. Depending on the immunobiological agent and the outcome measurement used, an absence of response can be expected in 25%–50% of cases.

**Table 3:** Evaluation of response to initial treatment (4–6 months) with immunobiologicals.

At least 2 criteria:
Improved sense of smell (at least 1 degree in olfactory classification, eg from moderate hyposmia to mild hyposmia)
Improved nasal congestion (at least 2 points on VAS)
Decrease in nasal polyp – decrease of 2 points in the Lund-Kennedy endoscopic score (sum of right and left side)
Asthma Control
SNOT-22 reduction ≥9

If the degree of response with the drug is considered acceptable for the patient, drug continuation is recommended and the patient is instructed to be reassessed at least every 6 months.

If disease control is not acceptable for the patient, the associated use of oral corticosteroids for a short period during treatment with immunobiologicals may help to optimize symptom control. Another alternative is the surgical procedure, which can be considered to reduce the polypoid tissue and the disease burden, so that the immunobiological can be more effective. In both cases, the continued use of the immunobiological agent is justified. If the patient persists with symptoms after the above strategies, he is considered as not responding, and the option would be to change the immunobiological or a new surgery.

## Considerations about immunobiologicals in Brazil

In Brazil, the use of immunobiologicals is already a reality for other specialties such as rheumatology, allergy, and pneumology, and it should be increasingly used in otorhinolaryngology, especially in type 2 CRSwNP patients. Issues such as the cost and possible financing by health insurance or public health system must still be addressed so that patients who are refractory to the currently available treatments can benefit from these drugs.

## Final comments

Severe uncontrolled CRSwNP, associated with comorbidities (asthma, AERD, severe dermatitis) has a negative impact on health-related quality of life. Although there is a significant unmet need for treatment in patients with severe and uncontrolled CRSwNP, the pharmacoeconomic justification for the use of immunobiological products is under development. Of particular interest for decision making will be the identification of these subgroups of patients refractory to the existing treatment options and the construction of a strategy that is clinically effective and improves their quality of life.

So far, the use of immunobiologicals can provide important benefits for these patients and it should be an evaluated, understood, and improved strategy in the future, with more studies and greater clinical experience.

## Conflicts of interest

The authors declare no conflicts of interest.
